# Modernizing CDC’s Practices and Culture for Better Data Sharing, Impact, and Transparency

**DOI:** 10.5888/pcd21.230200

**Published:** 2024-03-21

**Authors:** Jennifer L. Wiltz, Brian Lee, Rachel Kaufmann, Timothy J. Carney, Kailah Davis, Peter A. Briss

**Affiliations:** 1 National Center for Chronic Disease Prevention and Health Promotion, Centers for Disease Control and Prevention, Atlanta, Georgia; 2 US Public Health Service, Bethesda, Maryland; 3 Office of the Chief Information Officer, Centers for Disease Control and Prevention, Atlanta, Georgia

Making full use of data assets can allow institutions to support decisions, protect health, serve customers, and steward resources (1). Rendering data open to examination while protecting privacy and confidentiality may also enhance trust (1). Public health, research, and publication communities play key roles in data modernization through their work, partnerships, and leadership.

At the federal level, a comprehensive Federal Data Strategy (1) has been developed that provides a unified approach to data management, use, and sharing. Additionally, the *Foundations for Evidence-Based Policymaking Act* and the Presidential Memorandum on scientific integrity and evidence-based policymaking emphasize using data and evidence to inform decision making (2,3).

Government, including the Centers for Disease Control and Prevention (CDC), has embraced these changes. Work on this topic aligns with the larger CDC Moving Forward initiative and supports efforts to share science and data faster, translate findings into evidence-based policy, prioritize communications, promote results-based partnerships, and develop a prepared workforce — all to enhance trust and improve our impact on the lives of Americans and people around the world (4).

This article presents work that our organization, the National Center for Chronic Disease Prevention and Health Promotion (NCCDPHP) at the CDC, and CDC more generally have been doing to make data assets more broadly available. This article suggests data practices based on literature and CDC NCCDPHP experiences that can be valuable to the field, presents use cases and provides selected tools and supports, and promotes a culture that encourages good data practices (Figure). While our work will not apply seamlessly to all organizations and contexts, we are hopeful that lessons can be learned that might deserve broader application by the public health and scientific communities.

**Figure:**
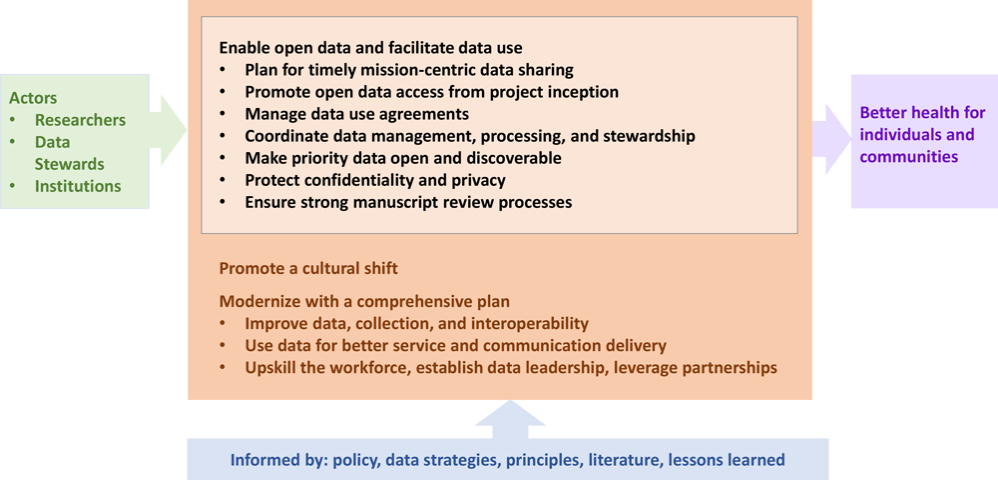
Data practices to amplify impact and promote transparency through the lifecycle (black, center) informed by what we know (blue, bottom) with changes achieved by actors (green, left) in the setting of broader modernized data science strategies (orange) that leverage data use to accomplish mission objectives (purple, right).

## Practices, Responsibilities, and Opportunities

Data sharing can be considered throughout project activities and requires support and collaboration among scientists, organizational leadership, data stewards, and others responsible for data management policy and practice. Consistently using effective data practices throughout the data lifecycle (5) can reduce the burden of managing data; increase use of data for multiple purposes; increase reuse and interoperability of data; improve reproducibility and replicability; and contribute to innovation and adoption of new methods, tools, and technology. Incorporating principles, such as FAIR (6) (Findability, Accessibility, Interoperability, and Reusability) and those offered in the Federal Data Strategy (1) (on ethical governance, conscious design, and learning culture), should serve as motivational guidelines for decisions, strategy, and informing practices.

Data sharing with the public and relevant partners is foundational to data modernization and an obligation of public health agencies at all levels. CDC policy requires us to manage and facilitate public access to publicly funded public health data, but any institution can operationalize and scale good practices for data.

With appropriate training and support, every practitioner can adopt good practices related to data management and sharing (7). Practitioners and institutions should determine what levels of access are appropriate for their data (ie, available publicly, available with restricted access, or unavailable to the public) and clearly communicate this access level.

The practices described (Figure) are intended as examples for data stewards and manuscript authors that might deserve wider use. Our intent is not to define all practices but to identify and recommend key fundamentals to enhance how data can be understood and reused, establish basic data hygiene standards that can be expected by users from shared data, and to meet privacy, ethics, and legal responsibilities. These practices should be tailored to local needs and capacities. Data practices for institutions and practitioners include the elements described in this article.

### Plan for timely mission-centric data sharing

Sharing data and science important to mission and partners starts with the evidence-building plan that identifies data needs for answering priority questions (1). Organizations should anticipate the data that are essential for decision making and public consumption and be prepared with the data analyses and products.

NCCDPHP is also working to share crucial data meaningful to partners and the public. As an example, Population Level Analysis and Community EStimates (PLACES) provides model-based estimates across 36 health measures, including 7 disability measures added in 2023, for every county, city, and census tract in the US (8). By collaborating with the Robert Wood Johnson Foundation and the CDC Foundation, we provide data that were previously rarely available for most geographic areas below the state level. PLACES leverages data collected through the Behavioral Risk Factor Surveillance System from more than 400,000 interviews across the US each year. The measures include major risk behaviors that lead to illness, suffering, and early death as well as the conditions and diseases that are the most common, costly, and preventable of all health problems. Growing from this partnership established in 2015, based on partnership feedback and planned improvements, these data now allow health departments and jurisdictions of all population sizes and rurality to better understand the health of their local populations and advance their public health mission. PLACES has typically released data annually within 12 months of availability. By intentionally prioritizing production, adding resources, starting processing earlier, and formalizing the quality control process, this year PLACES has released data 5 months early. Public interactive maps allow viewers to explore these key health-related measures, community by community, to plan programs and action that support their efforts to improve healthy life expectancy, quality of life, productivity, and health care costs.

### Promote open data access from project inception

Vetting information technology and data investments before activities begin can help ensure that a project is appropriately aligned with priorities and resources, relevant experts are involved, and data use practices are appropriate. At CDC, we conduct concept and investment reviews at the agency level and in CDC’s constituent Centers. These investment reviews help ensure that we follow good data practice (1) by establishing appropriate terms and conditions for contracts, grants, and other agreements to meet open objectives and data management requirements.

Early project development should also include consideration of partnerships and whether tools such as Memoranda of Understanding (MOU) or Memoranda of Agreement (MOA) involving data sharing can facilitate data use by defining the responsibilities for and acceptable use of data to be exchanged. When data cannot be shared in total (eg, because of privacy concerns) (9), practitioners ­should define what subsets or derived data are appropriate for release.

During the project planning phase, a data management plan (DMP) can be created and regularly updated as plans evolve. Any public health data set collected or generated using federal funds must have a DMP. The NCCDPHP template for this content contains provisions for accessing, standards for collecting, releasing, and preserving data long-term (10). NCCDPHP contract and grant awardees are expected to maintain a DMP and to make their federally funded data publicly available and discoverable, unless there is a compelling reason not to do so.

### Manage data use agreements

Data use agreements (DUAs) document the agreed plan for content and granularity of data sets that will be released. CDC launched an internal DUA repository, including more than 1,000 active signed DUAs from NCCDPHP, that serves as a reference for how these agency assets can be used and shared with partners.

A standardized DUA templating process can increase data use by avoiding unneeded data strictures and can mitigate organizational risks by ensuring content is included that is required by policy and law. CDC developed a template with input from partners that includes necessary federal language to engage in data sharing. The new process that allows staff to generate a DUA with standardized language in an electronic system also decreases burden and improves service delivery.

### Coordinate data management, processing, and stewardship

We identified practices from many organizations and authors (7,11–13) and adapted them in a checklist spanning 3 areas of data hygiene practices: 1) data management and organization; 2) code, software, and statistical processing practices for manipulation and analysis; and 3) collaboration (see Appendix for the full checklist integrated within the 7 overarching data sharing practices of this manuscript). In NCCDPHP, efforts span these areas of good data hygiene practices. As an example of good management, several systems (eg, Data Trends and Maps) are saving data as raw (Bronze), intermediate (Silver) and analytic ready (Gold) as they are migrated into an enterprise-wide data analytics and visualization platform. We hope that readers and their institutions might find this checklist or an adapted version useful in their organizations.

To establish practices in all 3 areas, the CDC COVID-19 Response convened data science experts and leaders to support open data using modern methods of data automation, computational privacy protection (14), stakeholder collaboration, and enhancements in accessibility to allow automated processing. Data formatted to be machine-readable (ie, structured to allow automated computer processing without losing meaning) are more interoperable than data that require human processing (eg, PDF) before use and reduce the burden on people needing to find and use data (2). Using these data hygiene practices allowed the CDC COVID-19 Response to design, verify, and release multiple data set iterations efficiently during the pandemic. 

As good processing practice, NCCDPHP developed the Publication Operations process (PubOps) to automate publishing of cleared data to the appropriate data repository. This automated data publishing pipeline reduces the timeline for data publishing from 6 months to a few weeks, reduces the complexity of data publishing process, and supports traceable data clearance processes, which all result in a more transparent process. Moreover, all NCCDPHP public-facing data visualization products have code saved in a source control repository such as github.com/CDCgov. The Study Tracking and Reporting System (STARS) is used at CDC to support the submission, review, clearance, tracking, and reporting of activities.

### Make priority data open and discoverable

To help users find and use open data, a comprehensive inventory of enterprise data assets is needed. Agencies must develop, publish, and update this inventory with the priority data sets for which disclosure is in the public interest (1,2). Agencies are to evaluate and improve the timeliness, completeness, consistency, accuracy, usefulness, and availability of open data assets. Agency data assets can then be shared in the US government’s data.gov open data catalog. Additionally, providing metadata describing the data asset makes data sets discoverable and easier to use. Data stewards should provide documentation and a data dictionary for all fields, in a machine-readable format where possible.

As of February 2024, NCCDPHP has made publicly available 224 data sets that are aligned with mission priorities, impactful, and useful to partners. We are providing standard technical and business metadata schema required for inclusion on data.gov and developing additional elements so that consumers have sufficient information (eg, to understand strengths, weaknesses, analytical limitations, security requirements, processing) relevant to use. Inclusion on data.cdc.gov will provide a one-CDC approach to the public for access to all CDC data.

### Protect confidentiality and privacy

Ensuring that authorities, roles, policies, and resources are in place is important to control access to confidential data and to safeguard privacy (1). Institutions can assess and apply data protection methods to strengthen privacy and confidentiality including: appropriately protecting or excluding private information when releasing data publicly, taking steps to mitigate the threat of re-identification of individuals and businesses if different publicly available data are combined, and training staff and modernizing data governance processes to support confidentiality and privacy design. NCCDPHP maintains a Data Release Review Committee to review planned data releases and protect those who provided the data. To do this, the Committee ensures that released data comply with informed consent and that personally identifiable information is removed. Also, potential to reidentify respondents with remaining information is assessed.

### Ensure strong manuscript review processes

Institutional and peer review processes need to be strong enough to cope with increasingly complex data, novel data sources, and more advanced data analysis. Additionally, data access, code, and analytics sharing can be promoted in the review and submission processes. Manuscripts that include use of data can be reviewed for following good data practices and ensure that data and related code are available along with manuscripts whenever possible. The target (7) would be to include access to the data, the build code that shapes and produces the analytical set, and the data analytic code on which publications are based so that results can be examined and replicated.

## Cultural Shift and Future Vision

Organizational leaders, scientists and data stewards can begin today to accelerate culture change that promotes long-term data good practices. Some examples might include launching a data modernization culture change campaign, incentivizing data sharing by incorporating open data in performance plans and yearly reviews, and showcasing successful examples of data usage.

### Modernize with a comprehensive plan

Enabling open data and accelerating data cultural change as described here are critical pieces of a robust, integrated approach to using data (Figure). In addition, coordinated efforts are needed that ensure data quality, utility, integrity, and objectivity, and advance data maturity, data standards use, and innovation. Most importantly, data must be used, as examples, for better service and communication delivery; to guide policy, planning, and operations decision making; and in interpreting and conveying the evidence-based messages, which will require leadership at many levels, ranging from chief data officers at federal agencies to frontline staff who serve as data ambassadors. They will also require general skill building of the public health workforce with the goals of increased data science knowledge throughout organizations and engaging a range of partners in collaborative approaches for improving data and its use.

The CDC Data Modernization Initiative outlines broad aspects of data modernization needed across the overall public health landscape and addresses the CDC Moving Forward imperative to advance core public health missions, consistently deliver public health information, and guide decision makers with timely data. Our NCCDPHP portfolio (15) advances these goals and includes data modernization projects, a Chronic Data Modernization Playbook, Communities of Practice, an innovation laboratory, and learning sessions. Improving trust, building on innovations, and learning lessons across the data lifecycle from curation to sharing have also been noted as crucial for priorities such as pandemic preparedness and global health (16,17).

### Moving forward

Activities are under way to modernize data practices for access, protection, and use in many organizations including CDC. In addition to the examples and material presented here, an online repository of resources is available which can promote progress in data practices throughout the federal government and beyond (18). Advancements in data sharing and use are models of long-term good practices. In addition, every success is a building block for further advancements promoting transparency, furthering trust, and amplifying positive health impact.

Accomplishing data sharing to fully leverage data as an asset will be transformational for how we conduct our work and use data to support decision making and effective public health action. Practices as provided in this manuscript and principles that government are required to adhere to are intended to be good for public health and therefore can be useful for all practitioners and institutions to consider. Making progress will require that a culture of data should be integrated as part of the organizational lifestyle. The ultimate goal of leveraging data assets and improving data use is to better individual and population health outcomes.

## Appendix

Checklist for data practices that enable open data and facilitate use, both overall and with a focus on management, statistical processing, and collaboration. This checklist can serve as a tool to assist practitioners and institutions in meeting good data practices (7,11–13) and can be considered in context of activities that promote a cultural shift and modernize with a comprehensive data strategy.

**Plan for timely mission-centric data sharing**

❑ Create evidence-building plan that identifies data needs for answering priority questions.

❑ Record lineage of data used; include location, version, and any transformation or manipulation performed.

❑ Establish quality metrics and perform data quality tests throughout the data management process.*
*

**Promote open data access from project inception**

❑ Establish investment review process to vet IT and data investments before activities begin.

❑ Describe data fields, including description, type, and specific vocabularies, where appropriate.

❑ Create metadata for data to improve discovery and use by others, including preferred means of accessing data, data quality characteristics (eg, representativeness, temporal, geospatial), and preferred citation.

**Manage data use agreements**

❑ Leverage a data use agreement repository to reuse existing and relevant data use agreements.

❑ Standardize the DUA templating process to facilitate inclusion of content necessary for policy, protection, appropriate use and sharing.

**Coordinate data management, processing, and stewardship **

*Data hygiene practices for data management and organization*
**
**

❑ Create analysis-friendly data with a record of how each result, including intermediate, was produced.

❑ Record how data are produced, describing all stages of data, including intermediate results, pre- and postprocessing steps, including workflow and data pipeline.

❑ Save raw, intermediate, and analytical-ready data used for analysis, tables, plots, and figures.

❑ Consistently code variables within analytical datasets, differentiate between null, unknown, and suppressed values.

*Code, software, and statistical processing practices for manipulation and analysis*

❑ Automate data retrieval, processing, transformation, privacy protection, and generation of data used for analysis. Avoid manual data processing as much as possible.

❑ Record all external programs and packages used, including exact versions for all data, analysis, reporting, and visualization.

❑ Record code used to create tables, plots, and figures.

❑ Develop and release custom developed code using an open-source license, whenever possible.

❑ Apply and record version control to all custom developed scripts used in publication (eg, git) within a CDC approved, durable location including changelogs and history.

❑ Include brief, explanatory comments within every program.

❑ Include brief, explanatory overview file within every project (eg, README.md) with description of purpose, installation, and operation.

❑ Deposit code in an appropriate repository that provides a persistent digital identifier; open source in a public repository; non-open in a secure repository.

*Collaboration practices for publication and ongoing data lifecycle changes *

❑ Collaborate with data stewards for review and correspondence prior to publication.

❑ Use a dataset's preferred point of contact for correspondence, do not contact underlying sources directly.

❑ Publish a manuscript on the dataset if the dataset is unique and available for others.

❑ Publish a data management plan where appropriate.

❑ Use preferred citations for each dataset that is used within the manuscript.

❑ Appropriately include data managers and data designers who contributed substantially to the data processing as authors or acknowledge within the manuscript.

❑ A draft version of the manuscript can be made available on a preprint server (eg, arxiv.org, www.bioarxiv.org, medrxiv.org).

❑ Data stewards can: a) Provide a method for users to report questions, potential errors, bugs, or defects in data and be responsive to user requests. At a minimum, email. Preferred, automated system allowing user to view status and link to dataset revision. b) Publish guiding material to assist users in understanding data, planning analyses, and using data. At minimum, a frequently asked questions document revised based on user need.

**Make priority data open and discoverable**

❑ Develop, publish, and update an inventory of data assets.

❑ Prioritize data assets for sharing that are aligned with priorities of, impactful for, and useful to partners and the public.

❑ Evaluate and improve the characteristics (eg, timeliness, completeness, consistency, accuracy) of open data assets.

❑ Supply metadata describing the data asset.

❑ Deposit data in an appropriate repository (eg, data.cdc.gov for all of CDC data) that provides a persistent digital identifier (preferably Direct Object Identifiers, or DOI), release data using an open format, and provide machine readable access to data and metadata. Put open data in a public repository; place non-open data in a secure repository.

**Protect confidentiality and privacy**

❑ Ensure authorities, roles, policies, and resources are in place to control access and safeguard privacy, eg, ensure adequate deidentification of the data, mitigate threat of reidentification, and support confidentiality and privacy design.

**Ensure strong manuscript review processes**

❑ Ensure capacity to review complex, novel data, and advanced analytics.

❑ Check for good data practices, eg, access to data, build code, and analytic code.
